# Identification and prediction of mixed-use functional areas supported by POI data in Jinan City of China

**DOI:** 10.1038/s41598-023-30140-x

**Published:** 2023-02-20

**Authors:** Minrui Zheng, Hongyu Wang, Yiqun Shang, Xinqi Zheng

**Affiliations:** 1 School of Public Administration and Policy, Renmin University of China, Beijing, 100872 China; 2 School of Information Engineering, China University of Geosciences (Beijing), Beijing, 100083 China; 3 College of Geoexploration Science and Technology, Jilin University, Changchun, 130026 China

**Keywords:** Socioeconomic scenarios, Sustainability

## Abstract

The urban development of China is changing from incremental expansion to stock renewal mode. The study of urban functional areas has become one of the important fundamental works in current urban renewal and high-quality urban development. In recent years, big spatiotemporal data has been well applied in the urban function field. However, the study of spatial–temporal evolution characteristics and forecasting optimization for mixed-use urban functional areas has not been examined well. Thus, in this study, we proposed a new approach that applies a revised information entropy method to analyze the degrees of mixing for urban functional areas. We applied our approach in Jinan City, Shandong Province as the study area. We used Point-of-Interest, OpenStreetMap and other datasets to identify the mixed-use urban functional areas in Jinan. Then, the CA–Markov model simulated the urban layout in 2025. The results showed that: (1) the combination of road network and kernel density method has the highest accuracy of identifying urban functional areas. (2)The mixing degree model is constructed by using the improved information entropy, which makes up for the shortcoming of identifying the mixed functional areas simply by the frequency ratio of POI data. (3) The “residence and business” functional area has the highest proportion in the central area of Jinan from 2015 to 2020, and the total area of mixed-use unban functional areas continuously increased during this period. (4) The total area of the central area in Jinan has significantly increased in 2025. The optimization of urban functions should expand mixed-use functional areas and increase the proportion of infrastructure. Also, Jinan should improve the efficiency of space development.

## Introduction

A city is a complex organism composed of its structure and functions. The urban spatial structure is the skeleton of a city, and urban functional layout is the concrete expression of the urban spatial structure. The urban development of China is gradually shifting from incremental mode to stock-based mode^1^, and the optimization and improvement of urban internal spatial structure and functions are one of the important topics in the field of urban renewal in the future^2^. In order to propose suitable governance strategies, it is necessary to identify current urban functions, and simulate and analyze different spatial structures of urban functions^3,4^.

The study of urban function is the basis of recognizing the complex urban system, exploring the change of urban land use, and realizing the sustainable development of the city. The research on identifying urban functional areas is conducive to a deeper understanding of urban spatial development. Also, the research on identifying urban functional areas plays an essential role in urban spatial planning and sustainable development decision-making^5^. Some scholars have examined well some parts of the urban functions, such as the identification of urban functional areas and evaluation of the current layout of urban functions. Some notable works are Chen et al.^6^ used Nanjing City as a study area to explore the similarity and differences between functional areas and administrative areas. They also analyzed the development history of urban functional areas and the trend of urban reorganization. Huang et al.^7^ provided an idea of sustainable development and five principles of urban zoning based on analyzing urban functional areas. Xing et al.^8^ examined the rationality of urban functional areas from the perspective of landscape patterns. Besides those works, some scholars also discussed the identification and prediction of mixed-use functional areas in western countries. For example, Song et al.^9^ reviewed a wide range of measures of urban land use mix and conducted a Monte Carlo simulation model to compare results from Hillsboro, Oregon, USA. They found that each measure provides a distinct perspective on mixed-use. Thus, the appropriate mixed-use measure should be determined by the number of land use dimensions of interest and the approximate scales at which land use mix influences the outcome of interest. Wandl and Hausleitner^10^ identified mixed-use functional areas in eight areas across Europe. In their work, they involved a number of different economic activities and the ratio of the working population to residential population within one area to describe mixed-use functions.

However, due to historical reasons and the formation characteristics of urban functional areas, a single functional area per each land use category (e.g., residence only in the residence category) is not suitable for the demand of current urban development. The reason is that single functional areas could cause extensive land use and low utilization of land use. From the urban renewal process in western countries, the construction of mixed-use functional areas has become the focus of urban renewal and development^11^. However, the urban planning in China has been focusing on single-use functions for a long time and not paying enough attention to mixed-use functions. In fact, during the development and evolution of cities, a variety of mixed-use functions areas have been formed, such as high-rise buildings, which may contain residential, office, and commercial services. In the high-quality development of Chinese cities, the emphasis on mixed-use functions is the national demand in the future. Facing the urgent purpose of urban renewal in modern society, identifying mixed-use functional areas, excavating the modes of mixed-use urban functions, and evaluating the rationality of mixed-use functions have become vital scientific problems^12^. Those issues should be solved first, then decision-makers can make a better decision and then improves the sense of gain, security, and happiness of residents.

With the development and maturity of new technologies, identifying urban functional areas involving big spatiotemporal data has been a hotspot topic in recent years. Big spatiotemporal data contains traditional geospatial data (with the advantages of high precision, wide coverage, and timely update). It also contains new types of geospatial data, such as cellular signaling data, social media data (e.g., Weibo Check-in), and GPS track records, which can provide more insights into urban management and territorial space planning^13^. In existing studies of urban functions, the commonly used big data includes POI data, mobile phone signaling data, social media data, and GPS data etc. Because of the features of POI data, close to land use data, crowdsourcing data characteristics, easy to obtain, each record includes name, category, coordinates, classification code and so on, more than 70% of those studies use POI data^14–18^. POI data has become the core data for location-based services^17^.

However, existing studies focused on using big spatiotemporal data to identify the boundaries of urban or main functional areas. Although some pioneers have already investigated the identification of mixed-use functional areas, these studies have not comprehensively examined identifying mixed-used functional areas^19^. For example, Zhao et al.^20^ compared ten measurement models of mixing degrees. They found that models are suitable for measuring the overall mixing degree at mesoscale, such as district and block. Li et al.^21^ explored the residential planning mode of mixed-use land use. Hu et al.^22^ examined the recognition of urban functional areas and their mixing degree in Chongqing using POI data. Li et al.^23^ combined POI and taxi-OD data to evaluate the mixed urban functions quantitatively. Moreover, they revised the information entropy model to develop spatial entropy and temporal entropy. Li et al.^24^ typically applied random forest to determine the weights of POI, then they used the urban function calculation model to identify single dominant functional areas and information entropy to calculate the mixing degree of urban functions. Although some scholars investigated the identification of mixed-use functional areas and their mixing degree, the identification and analysis of mixed-use urban functions are not in-depth. The major reasons are different classification standards, unclear definitions of mixed-use function units, and different spatial scales.

Furthermore, some scholars suggested that the study of the identification of urban functional areas should use multi-source data. Different levels and functional areas should use different datasets and identification units. These suggestions showed that the identification of urban function areas can improve accuracy and efficiency through new technologies and multi-source data and also need to create different identification processes and approaches for different levels and different functional areas. But, there is still no breakthrough in selecting appropriate functional identification units and approaches^25^. Because it is difficult to quantify the correct mix or the spatial relations among different POI types indicative of specific urban functions^26^.

We summarizde existing issues in the current urban functions field as follows: (1) there is a lack of comparative study on the approaches and identification units of different functional areas. (2) A personalized model for identifying urban mixed-use functions is lacking. (3) there is a lack of multi-level spatial analysis and optimization simulation analysis of urban function. Therefore, in this study, we adopted spatial grid and road network division units to identify Jinan functional areas using frequency density, category ratio and kernel density estimation method. Then, we evaluated the accuracy of our approach and found the most suitable identification approach for Jinan City. Also, we analyzed the spatial structure of Jinan's functional areas from 2015 to 2025 based on the CA–Markov model, and gave appropriate suggestions to optimize the layout of urban spatial structure. The approaches used in this study could provide a reference for the high-quality development of the city.

## Materials and methods

### Study area

Jinan City is the capital of Shandong Province and is famous for its spring city. It is also the political, economic, cultural, technological, educational and financial center of Shandong Province. Jinan City is located between 36°01′N-37°32′N and 116°11′E-117°44′E. Until 2020, Jinan has 12 county-level administrative regions, including ten municipal districts and two counties. They are Shizhong district, Lixia district, Huaiyin district, Tianqiao district, Licheng district, Changqing district, Zhangqiu district, Jiyang district, Laiwu district, Gangcheng district, Pingyin county, and Shanghe county. The total area of the city is 10,244km^2^, the total area of the built-up region is 760.6km^2^, and the urbanization rate is 71.21%. According to the latest census data, the registered population of Jinan reached 8.16 million, which ranked 27th in the country.

**Figure 1:**
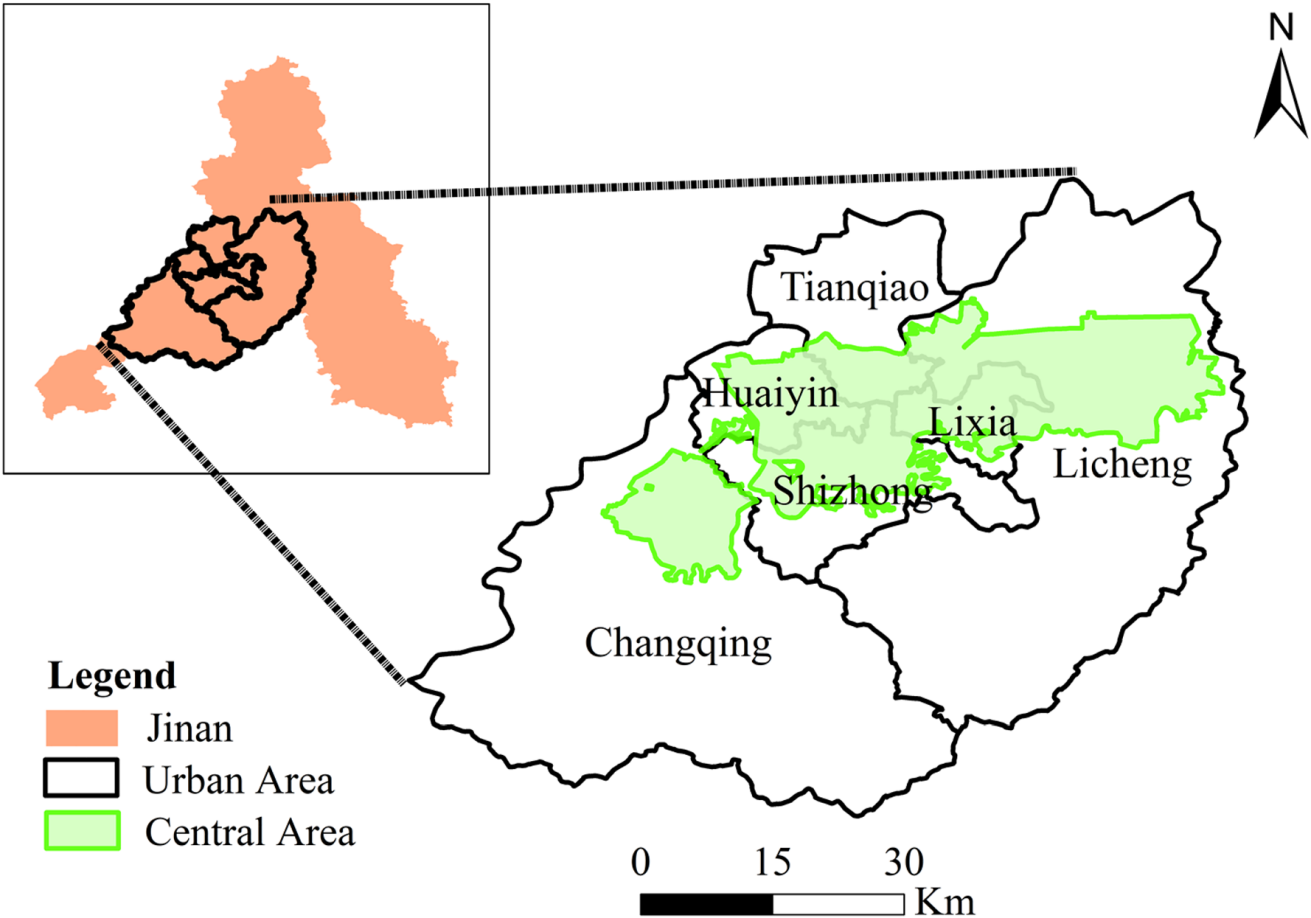
The map of Jinan City. The figure was created using ArcGIS 10.2 (https://support.esri.com/en/Products/Desktop/arcgis-desktop/arcmap).

Jinan connects Beijing-Tianjin-Hebei region in the north, and Yangtze River Delta in the south. It is one of the important transportation hubs in East China. Because of springs, Jinan is a famous historical and cultural city and one of the first batch of excellent tourist cities in China. Jinan is also a national pilot city for the transformation of new and old kinetic energy^27^. Thus, in the first three quarters of 2022, the GDP of Jinan (864.201 billion Chinese yuan) ranked 20th in entire China. In this study, we choose the downtown area and central area of Jinan as the study area, the map of this study is shown in Fig. 1.

### Data sources

*OpenStreetMap (OSM) Data*. OSM is one of road network data. It is easy to access and free for the public. OSM has high positioning accuracy and accurate topological relationship, the data includes longitude and latitude, road type, road name, maximum driving speed, and other attribute information. We downloaded the data from OSM website (https://www.openstreetmap.org/), and processed the data using ArcGIS software.

*Point-of-Interest (POI) Data*. POI data is the point data of real geographical entities with spatial attribute information. Meanwhile, it has a number of advantages, such as large data size, wide coverage, easy to obtain, detailed information (e.g., names of buildings, categories of buildings, and coordinates) and timely updates. Because of those advantages, POI data is a widely used dataset in urban studies. Currently, there are a set of available POI resources, such as Baidu, Gaode and Tencent in China, as well as Google and OSM in western countries. In order to provide an accurate and convincing result, we used Gaode POI data because it has better data integrity in our study area. In this study, we collected 2020 POI data from Gaode (https://maps.gaode.com/). Each POI record contains a series basic contents, for example, name, longitude and latitude, administrative region, and address.

The original POI data has three levels of categories (23 big categories, 267 mid categories, and 869 subcategories; https://lbs.amap.com/api/webservice/download). We removed duplicate and missing records. Finally, 72,323 records in 2015 and 332,329 records in 2020 were obtained. Based on “Standards of Urban Land classification and Planning Construction Land” (GB50137-2011) and land use classification of Jinan City, we further reclassified the data into six new categories, including residential area, business area, industrial area, public service facilities, traffic facilities, green space and square (Table 1)^28^.

**Table 1:** Information of POI data.

Categories	Number of records		Percentage (%)	
	2015	2020	2015	2020
Residential	4128	8542	5.71	2.57
Business	25,005	209,917	34.57	63.17
Industrial	27,521	34,354	38.05	10.34
Public services	9815	61,088	13.57	18.38
Traffic	3341	16,848	4.62	5.07
Green space	2513	1580	3.47	0.48
Total	72,323	332,329	100	100

*Other Datasets* Besides OSM and POI, we involved other datasets in verifying our results. The datasets include (1) boundary map of administrative zones. The boundary map of administrative zones of Jinan City was obtained from the Geographical Information Monitoring Cloud Platform (http://www.dsac.cn/). (2) Remote sensing images. We collected the remote sensing images from Google Earth and Gaode in August 2015 and May 2020, the spatial resolution of those images is 30 m × 30 m. (3) Statistical yearbook data. We acquired those datasets from the Jinan Bureau of Statistics (http://jntj.jinan.gov.cn/col/col27523/index.html). (4) Map of urban planning in Jinan.These maps mainly come from the related data of Jinan city government's official website (http://gh.nrp.jinan.gov.cn/).

### Research methods

*Frequency Density and Ratio Index*. Frequency density (FD) is the most commonly used index in the study of the identification of urban functions. This index gives the frequency per unit for the data in this class, where the unit is the unit of measurement of the data. In this study, FD is used to calculate the total records of POI data in each identification unit and then get the frequency density and ratio index per unit. For each functional area unit, we identify the functional areas based on frequency density and ratio index. The formulas are^29,30^:

1 $$F_{i} = \frac{{n_{i} }}{{N_{i} }}, i \in \left( {12...k} \right)$$

2 $$C_{i} = \frac{{F_{i} \times W_{i} }}{{\sum\limits_{i = 1}^{k} {\left( {F_{i} \times W_{i} } \right)} }} \times 100\% i \in \left( {1,2,...,k} \right)$$

where *i* indicates the type of POI, *k* is the total number of types of POI. *n*_*i*_ represents the number of type *i* POI in a unit, whereas *N*_*i*_ is the total number of type *i* POIs. *F*_*i*_ is the frequency density of type *i* POIs in a unit, and *C*_*i*_ is the proportion of the frequency density of type *i* POIs in a unit. *W*_*i*_ represents the weight for each frequency density (the general value is 1).

*Kernel density estimation (KDE) Method*. Kernel density estimation is the most important and intuitive method to measure the aggregation degree of POI data^30^. It can not only clearly display the number of POI records in each unit, it also can compare the aggregation degree of POI records in different areas, and further visualize the results^32,33^. The calculation of kernel density estimation is shown in Eq. (3):

3 $$f\left(x\right)={\sum }_{i=1}^{n}\frac{1}{{\mathrm{h}}^{2}}\Phi \left(\frac{x-{c}_{i}}{\mathrm{h}}\right)$$

where *f(x)* is the estimated kernel density at *x*. *c*_*i*_ is the *i*th spatial location of POIs within the bandwidth. *h* is the threshold of distance decay, i.e., bandwidth. *n* is the number of POIs that the distance between *x* is less than or equal to *h*. *Φ* is a spatial weight function. In this study, we use the quartic weight equation (Eq. 4).

4 $$\Phi \left( {\frac{{x - c_{i} }}{h}} \right) = \frac{3}{4}\left[ {1 - \frac{{(x - c_{i} )^{2} }}{{h^{2} }}} \right]$$

*CA–Markov Model*. A CA–Markov model is a robust approach to spatial and temporal dynamic modeling of land use changes. Because the CA–Markov model absorbs the benefits from time series and spatial predictions of the Markov and CA theory, it can be used to carry out the spatial–temporal pattern stimulation^34,35^. There are a series of studies that used CA–Markov model, please see^35–37^ for more detail about this model. In this study, we use Markov model to get land transfer area matrix in IDRISI software, use LOGISTICREG module to generate suitability maps, and use CA–Markov model to simulate land use patterns in the future^38^.

*Information Entropy*. Shannon proposed information entropy in 1984. It is mainly a measurement of uncertainty, which describes the order degree of urban spatial structure. The value reflects the number of types of functional areas and the evenness of the distribution of different types of function areas. The more types of functional areas, the greater value of information entropy, and the lower the order degree of the functional area system. The equation is shown in Eq. (5)^39,40^:

5 $$H = - \mathop \sum \limits_{i = 1}^{n} \frac{{A_{i} }}{{\mathop \sum \nolimits_{i = 1}^{n} A_{i} }}\ln \left( {\frac{{A_{i} }}{{\mathop \sum \nolimits_{i = 1}^{n} A_{i} }}} \right)$$

where *H* is information entropy (unit: Nat), *n* is the total types of urban functional area. *A*_*i*_ is the total area of each type, and *A* is the total land area.

Based on the equation of information entropy, we can define the equilibrium degree of spatial structure:

6 $$J = \frac{H}{{H_{\max } }} = \frac{{ - \mathop \sum \nolimits_{i = 1}^{n} \frac{{A_{i} }}{{\mathop \sum \nolimits_{i = 1}^{n} A_{i} }}\ln \left( {\frac{{A_{i} }}{{\mathop \sum \nolimits_{i = 1}^{n} A_{i} }}} \right)}}{\ln n}$$

where *J* is the equilibrium degree, H is the actual information entropy, and H_max_ is the maximum information entropy. When J = 0, the spatial structure is in the most unbalanced state; When J = 1, the spatial structure reaches the ideal equilibrium state. In the meantime, dominance degree (J) reflects the degree to which one or multiple types of functional areas dominate the region. It is opposite to the equilibrium degree. The equation is shown in Eq. (7).

7 $$I=1-J$$

*Mixing Degree Model*. The mixed-use urban functions typically represent different functions (two or more functions) within a unit. A typical example is commercial (lower stories are used for commercial functions) and residential (higher stories are used for residential functions) buildings. In order to address the shortcomings of FD, we apply revised information entropy to measure the degree of mixing for urban functions. We adopt ratio index into the conventional information entropy, which overcomes the shortcoming that determines the mixed-use urban functions only depending on FD and ratio index. The high value of revised information entropy stands high degree of mixing (more functions for one unit)^41^, the equation of revised information entropy is:

8 $$M = - \sum\limits_{i = 1}^{n} {\left( {C_{i} \times \ln C_{i} } \right)}$$

where *M* is the degree of mixing. *n* is the number of POI types in one unit, *C*_*i*_ is the ratio index of POI types in one unit.

## Results and discussions

### The selection of identification of urban functional areas approach

#### The approaches of identification of urban functional areas

In this study, we compared the performance among three identification approaches: (1) spatial grid and FD, (2) OSM, KDE and FD, and (3) OSM and FD. We also did mutual verification experiments among those three approaches.

The spatial grid and FD approach first divides the research area into grid units with fixed unit size. Based on our previous work, we empirically use 50 m × 50 m grid cells. Then POI data is involved in calculating FD and ratio index per grid. When two-category functional areas have larger Ci values (20% ≤ Ci ≤ 50%) in one grid unit, this grid unit is assigned to a mixed-use functional area. When only one functional area has larger Ci values (Ci ≥ 50%), this grid unit is assigned to a single functional area. When Ci equals 0, this grid unit is no data area. The rest situations are integrated areas.

The OSM, KDE and FD approach divides the whole study area into units based on road network, and then KDE calculates the density based on POIs. We also involve FD and ratio index to group the identification units. According to the classifications of functional areas in Jinan, we use 200 m as the bandwidth of KDE. The standard of classification of FD and ratio index is the same as the spatial and FD approach.

The OSM and FD approach can be seen as a combination of previous two approaches. That is, the identification unit is divided based on the road system, and the identification results are calculated by FD. For detailed information, please refer to spatial grid and FD approach and OSM, KDE and FD approach.

#### Results of identification of urban functional areas

We use confusion matrix and kappa coefficient to evaluate the performance of each approach. Typically, the range of kappa coefficient is between 0 and 1. The higher value of kappa coefficient, the result is more accurate. The kappa coefficient value is between 0 and 0.2, the results have the lowest accuracy. When the value of kappa coefficient is between 0.41 and 0.6, the results are acceptable. The value between 0.61 and 0.8 stands for higher accuracy, whereas the value is greater than 0.81, the results have the highest accuracy.

The confusion matrix result depends on remote sensing images of Jinan in 2020 from Google Earth and Gaode. We randomly selected 40 identification units to calculate the confusion matrix and kappa coefficient. Table 2 shows the overall accuracy and kappa coefficient for each approach.

**Table 2:** Overall accuracy and kappa coefficient for each approach.

Spatial grid + frequency density		OSM + kernel density estimation + frequency density		OSM + frequency density	
Overall accuracy	Kappa coefficient	Overall accuracy	Kappa coefficient	Overall accuracy	Kappa coefficient
77.63%	0.71	80.83%	0.77	73%	0.67

Comparing overall accuracy and kappa coefficient, the OSM, KDE and FD approach has the higher accuracy, which overall accuracy is around 80% and kappa coefficient is around 0.77. Thus, we choose this approach to identify the urban functional areas in this study. The confusion matrix is shown in Table 3.

**Table 3:** Confusion matrix of OSM, KDE and FD approach.

Identified Reference	Residential area	Business area	Industrial area	Public service facilities	Road traffic facilities	Green space and square	#units	Producer accuracy (%)
Residential area	**30**	2	3	3	1	1	40	75%
Business area	2	**34**	1	1	2	0	40	85%
Industrial area	2	2	**28**	3	3	2	40	70%
Public service facilities	1	1	1	**36**	1	0	40	90%
Road traffic facilities	0	2	3	1	**32**	2	40	80%
Green space and square	0	1	0	2	3	**34**	40	85%
#units	35	42	36	46	42	39	**240**	
User accuracy(%)	85.71%	80.95%	77.78%	78.26%	76.19%	87.18%		

Significance values are in bold.

### Characteristics of Jinan functional areas

#### “Residential/Business” is the highest proportion of mixed-use functional areas

In 2020, the downtown area of Jinan City has 13,113 units based on the road system division, and the total area is 3254.72 km^2^ (Table 4). There are 2700 units in the central area with a total area of 651.02km^2^. For single functional areas, residential areas are the largest functional areas in the downtown area and central area of Jinan. The total residential areas in downtown and central areas are 324.16km^2^ (10.28%) and 225.35km^2^ (34.62%). For mixed-use functional areas, residential/industrial (538.36km^2^) and residential/business (105.40km^2^) areas have the largest area in downtown and central area, respectively. Those results are consistent with the actual situation.

**Table 4:** The identification result of Jinan City.

Type	Downtown area (km^2^)	Percentage (%)	Central area (km^2^)	Percentage (%)
Residential	324.16	10.28	225.35	34.61
Residential/Industrial	538.36	17.07	115.46	17.74
Residential/Traffic	77.56	2.46	2.26	0.35
Residential/Green Space	125.60	3.98	3.70	0.57
Business	17.50	0.55	4.72	0.73
Business/Residential	252.62	8.01	105.40	16.19
Business/Industrial	77.87	2.47	21.48	3.3
Business/Traffic	2.69	0.09	1.80	0.28
Business/Green Space	0.49	0.02	0.49	0.08
Industrial	259.29	8.22	69.21	10.63
Industrial/Green Space	43.05	1.36	4.24	0.65
Public Services	33.44	1.06	2.25	0.35
Public Services/Residential	132.32	4.19	11.56	1.78
Public Services/Business	231.91	7.35	19.80	3.04
Public Services/Industrial	181.10	5.74	5.72	0.88
Public Services/Traffic	187.01	5.93	7.24	1.11
Public Services/Green Space	2.77	0.09	0.16	0.02
Traffic	16.93	0.54	3.10	0.48
Traffic/Industrial	18.99	0.6	1.11	0.17
Traffic/Green Space	71.29	2.26	0.33	0.05
Green Space	156.59	4.96	7.27	1.12
Integrated Area	401.52	12.73	37.84	5.81
Others	1.66	0.05	0.53	0.08
Total	3254.72	100	651.02	100

**Figure 2:**
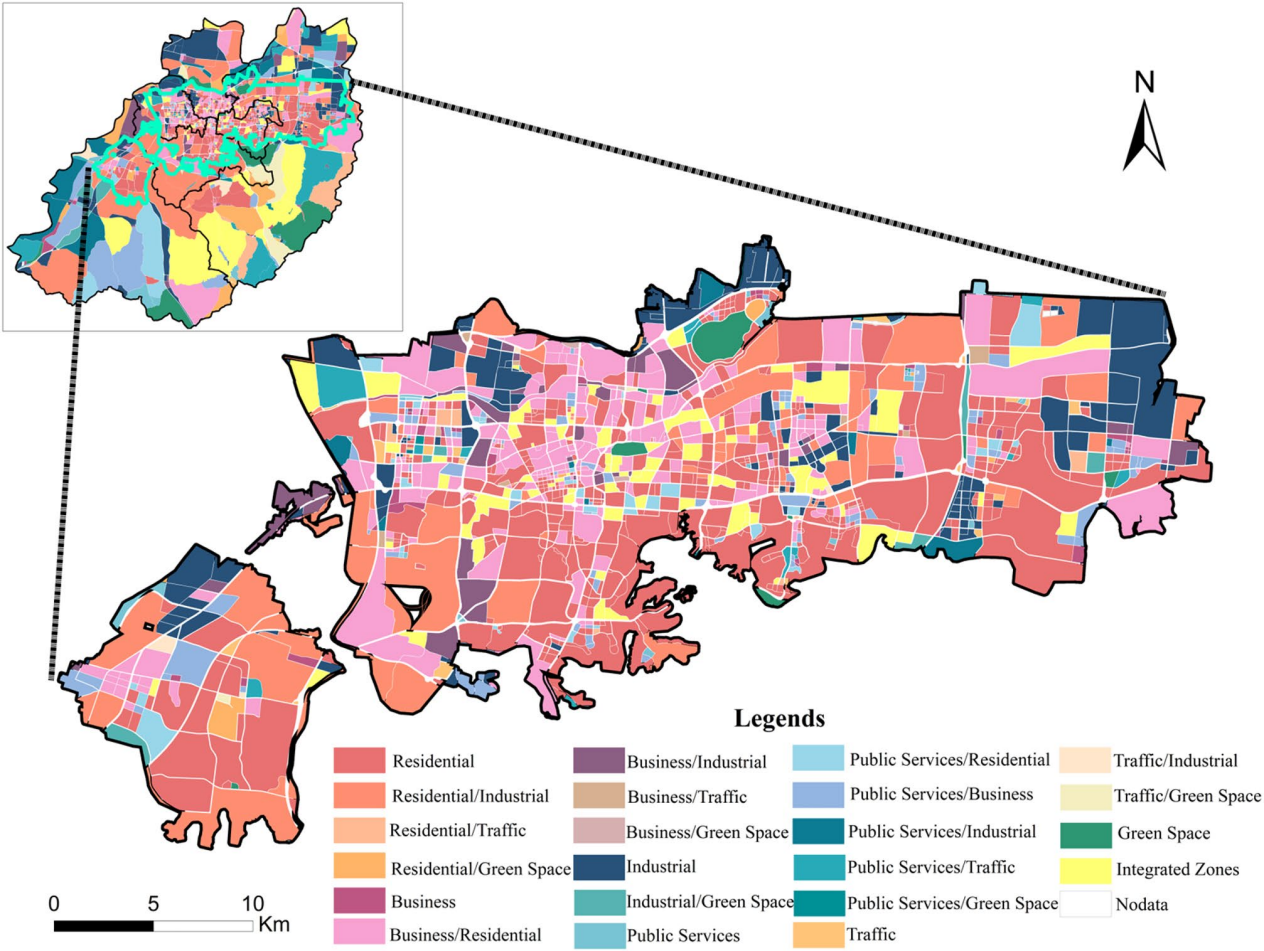
Identification results of urban functional areas in central area of Jinan City. The figure was created using ArcGIS 10.2 (https://support.esri.com/en/Products/Desktop/arcgis-desktop/arcmap).

The spatial distribution of urban functional areas in Jinan City can be found in Fig. 2. Residential and business areas are mainly distributed in the central area. Industrial areas are far from the central area. Public service facilities and green spaces distribute sporadically. As discussed in Fan et al. and Dou^42,43^, they also found similar overall characteristics as ours. Fan et al.^42^ used kernel density analysis method and Gaode POIs data to study the functional zoning structure of Jinan city. Dou^43^ used multiple spatial analysis methods and models based on multi-source data such as POI data, remote sensing images, road network data, Baidu heat map and traditional statistical data. Although we used different methods and data sources, the findings are consistent. We found that the urban functional spatial structure of Jinan city has a significant agglomeration characteristic of residential and commercial function elements, and the industrial production function elements transfer to the periphery of the city.

#### The mixed-use functional areas in the downtown and central areas have been increasing

**Figure 3:**
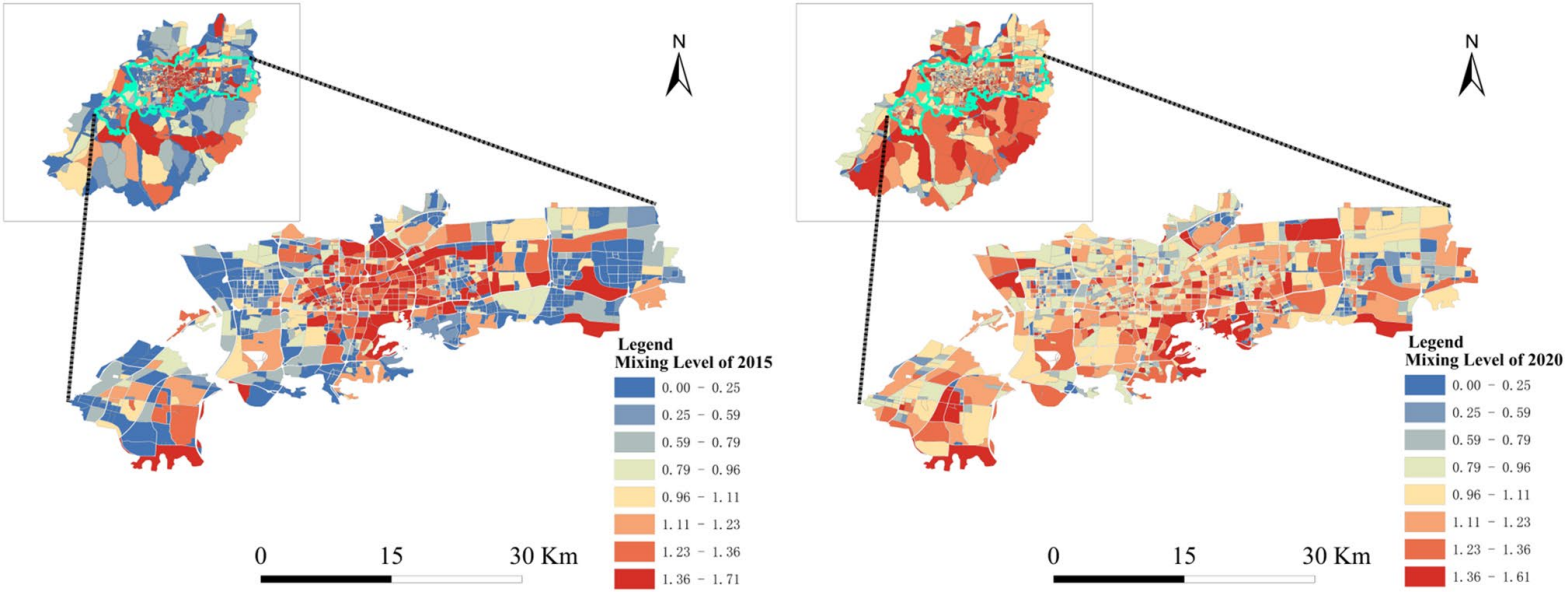
Mixing degrees of downtown and central area in Jinan in 2015 (left) and 2020 (right). The figure was created using ArcGIS 10.2 (https://support.esri.com/en/Products/Desktop/arcgis-desktop/arcmap).

The mixing degree of urban functions in the downtown and central areas has been increasing from 2015 to 2020 (Fig. 3). Yu et al.^44^ also found that the urban functional areas of Jinan City have prominent features of mixed functional areas. In 2015, the mixing degree of urban functional areas showed a trend that diffuses from the center to the edge of the city. In 2020, the entire central area has a higher mixing degree. This finding indicates that the development of the central area in Jinan City is becoming more and more consummate.

The degree of urban function recombination refers to the mixed state of urban functions. In other words, the state where two or more urban functions are mixed in a specific space or time range^45^. From the analysis of the evolution characteristics of urban spatial structure and mixing degree, the values of degree of urban function combination in Jinan were unstable from 2015 to 2020 (Table 5). Moreover, equilibrium degrees slightly decreased in both downtown area and central area. On the other hand, dominance degrees showed a slightly increasing trend. Those results demonstrated that the spatial distribution of urban functional areas in Jinan had reached a relatively reasonable equilibrium state. Our result is consistent with Reference^42,43^.

**Table 5:** Results of information entropy, equilibrium degree and dominance degree of urban functional areas in Jinan City.

Index	2015		2020	
	Jinan City	Central city	Jinan City	Central city
Mixing degree M	1.49	1.47	1.46	1.45
Equilibrium degree J	0.83	0.82	0.81	0.80
Dominance I	0.17	0.18	0.19	0.20

### Prediction and optimization suggestions of urban functions in Jinan City

#### Prediction results of urban functional areas

**Figure 4:**
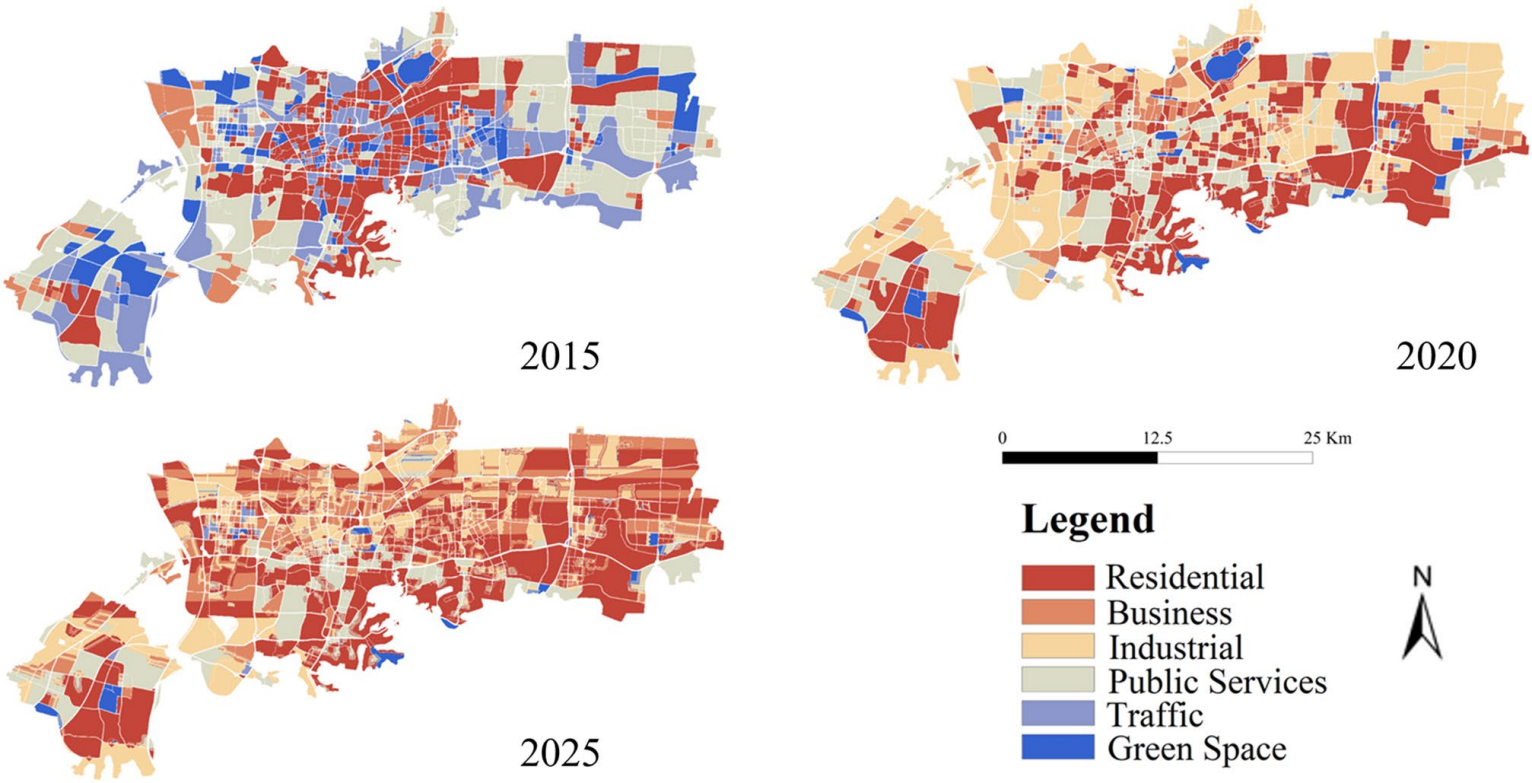
Spatial and temporal distribution patterns of urban functional areas in Jinan City from 2015 to 2025. The figure was created using ArcGIS 10.2 (https://support.esri.com/en/Products/Desktop/arcgis-desktop/arcmap).

In this study, based on identified ​results of the combination of road network and kernel density, we used CA–Markov model to simulate the urban functional areas of Jinan City in 2025. The entire predictive process is implemented in IDRISI software(https://clarklabs.org/)^46^. CA–Markov model is widely used in the prediction of land use land change, and is a relatively mature model. Because of the dispersibility and randomness of mixed-use functional areas, our predictive model was based on single functional areas. Therefore, for mixed-use functional areas, the function with the highest proportion was assigned to the closest single functional area, for example, 55% of residential/business functional area is a residential area, and this area will assign to the residential area. The spatial and temporal distribution patterns of urban functional areas in Jinan City is shown in Fig. 4.

From Fig. 4, the number of residential areas (tangerine) and business areas (orange) shows a significantly increasing pattern, but ancillary facilities (e.g., public service facilities (green), traffic facilities (light blue), green space and square (dark blue)) of those areas do not have the similar pattern as residential and business areas. Thus, the number of ancillary facilities should increase in the future. Jinan City needs to pay more attention on service convenience and urban landscaping, which could improve the degree of living comfort and happiness index of residents.

As one of the traditional industrial cities, the layout of industrial areas in Jinan City shows three development paths: distributed sporadically in the entire central area in 2015, clustering in the central area in 2020, and distributed on the edge of the central area and having block-based development mode in 2025. These results indicate that Jinan has been adjusting industrial layout to achieve high-end industrial development, compact spatial layout, intensive land use, and strengthening the key industries.

#### Suggestions for urban functional areas in Jinan City

According to our results, we have a number of suggestions for urban renewal in Jinan City. The suggestions are:

1. Jinan has a smaller area of green space and square, larger area of business areas, lower per capita traffic facilities area, and lower per capita green space and square area. Therefore, It is necessary to increase the proportion of mixed-function land, improve the level of land intensive use, and ensure the sustainable development of land resources.
2. The layout of business areas is dispersed, and there is still room for improvement in the degree of aggregation of business areas in Jinan City. Under the current urban structure, it is not only increasing costs of urban construction but also not conducive to intensive land use and sustainable development in the future. Therefore, gradually adjusting the spatial structure of urban functional areas is necessary. In particular, residential/industrial functional areas need to be adjusted because it affects the living environment.
3. According to our findings, we suggest that residential and business areas will continue to expand in the future. At the same time, the number of public service facilities, road traffic facilities, green space and square, and other infrastructure facilities may be reduced correspondingly. Jinan City should replan urban development strategies in order to satisfy the needs of residents.
4. Decision-makers should consider the relationship between the intensity of spatial development and regional planning, use different planning policies in different areas, promote intensive land use development, and promote simultaneous development of urban and rural areas.
5. Jinan City should increase the proportion of mixed-use functional areas and infrastructure facilities, improve the efficiency of urban space development, and achieve the maximum value of land use with the less occupied area^47^.

## Conclusion

This study compared three identification approaches to identify urban functional areas in Jinan City based on POI data. Meanwhile, confusion matrix and kappa coefficient to verify the accuracy of each approach. This study also analyzed the urban spatial structure of Jinan City, summarized existing issues in the structure of urban functions in Jinan, and provided a number of suggestions to adjust the spatial structure of Jinan in the future. The primary conclusions of this study are as follows:

1. This study compared three identification approaches: (1) spatial grid and FD, (2) OSM, KDE and FD, and (3) OSM and FD. They all perform well, but the OSM, KDE and FD approach perform better than others.
2. From 2015 to 2020, the total area of mixed-use functional areas was continuously increasing. Therefore, the distribution of functional areas in downtown and central areas is rationality.
3. In 2025, residential and business areas will continually expand, but their related ancillary facilities, such as road traffic facilities and green space, cannot satisfy residents’ needs.
4. In order to optimize the spatial layout of urban functions in the future, Jinan should reasonably expand the proportion of mixed-use functional areas and infrastructure land and improve the efficiency of urban space development and utilization.

Our future work will focus on a set of trends. First, we only involved POI data in this study. We will use more datasets in the future to compare their performance. Second, although the OSM, KDE and FD approach achieved the highest accuracy in this study. The limitations of OSM and POIs (e.g., uneven distribution of data) may cause bias. Thus, we will apply multiple road network datasets to examine the results. Third, our simulation model only had two historical datasets. We will use more historical datasets and different simulation models to do a contrastive analysis. In addition, a gold standard/benchmark to validate functional areas and associated uses should be examined in the future.

## Data Availability

The datasets used and/or analyzed during the current study are not publicly available due to General Data Protection Regulations; however, they are available from the corresponding author upon reasonable request.
